# Serum growth differentiation factor 15 as a biomarker for malnutrition in patients with acute exacerbation of chronic obstructive pulmonary disease

**DOI:** 10.3389/fnut.2024.1404063

**Published:** 2024-07-10

**Authors:** Guifen Shi, Longfei Yue, Zhengying Tang, Yingling Wang, Xiwei Hu, Yufeng Tong

**Affiliations:** ^1^Department of Respiratory and Critical Care Medicine, The Affiliated Hospital of Guizhou Medical University, Guiyang, China; ^2^Department of General Medicine, The Anshun People’s Hospital, Anshun, China; ^3^Department of Respiratory and Critical Care Medicine, The Non-directly Affiliated Anshun Central Hospital, Guizhou Medical University, Anshun, China

**Keywords:** chronic obstructive pulmonary disease, acute exacerbation, growth differentiation factor 15, malnutrition, biomarker

## Abstract

**Background:**

Chronic obstructive pulmonary disease (COPD) is a common respiratory disease that often coexists with malnutrition during acute exacerbation (AECOPD) and significantly affects the prognosis. Previous studies have shown that growth differentiation factor 15 (GDF15) levels promote appetite suppression, weight loss, and muscle weakness, and are markedly high in peripheral blood following inflammatory stimulation. However, it is still unknown whether serum GDF15 levels can be used to predict malnutrition in patients with AECOPD.

**Methods:**

A total of 142 patients admitted to the Department of Respiratory Medicine at Anshun People’s Hospital between December 2022 and August 2023 were selected for this study. The participants were divided into two groups: malnutrition group (*n* = 44) and non-malnutrition group (*n* = 98) based on a body mass index (BMI) < 18.5 kg/m^2^, according to the Global Leadership Initiative on Malnutrition (GLIM) criteria. Serum GDF15 levels were measured using the enzyme-linked immunosorbent assay (ELISA) and compared between the two groups. Spearman correlation analysis was used to examine the association between serum GDF15 levels, baseline data, and clinical indicators. Binary logistic regression was used to identify the independent risk factors for AECOPD combined with malnutrition. The predictive value of serum GDF15, albumin (ALB), and a combination of these was evaluated to identify malnutrition in patients with AECOPD using a receiver operating characteristic (ROC) curve.

**Results:**

Serum GDF15 levels in patients with malnutrition and AECOPD were significantly higher than those in patients without malnutrition, whereas the serum ALB levels were significantly lower than those in patients without malnutrition (*p* < 0.001). Moreover, serum GDF15 levels were negatively correlated with BMI (*r* = −0.562, *p* < 0.001), mid-arm circumference (*r* = −0.505, *p* < 0.001), calf circumference (*r* = −0.490, *p* < 0.001), total protein (*r* = −0.486, *p* < 0.001), ALB (*r* = −0.445, *p* < 0.001), and prognostic nutritional index (*r* = −0.276, *p* = 0.001), and positively correlated with C-reactive protein (*r* = 0.318, *p* < 0.001), COPD assessment test score (*r* = 0.286, *p* = 0.001), modified medical research council classification (*r* = 0.310, *p* < 0.001), and global initiative for chronic obstructive pulmonary disease grade (*r* = 0.177, *p* = 0.035). Furthermore, serum GDF15 levels were an independent risk factor for malnutrition in patients with AECOPD (OR = 1.010, 95% CI, 1.003∼1.016). The optimal cut-off value of serum GDF15 level was 1,092.885 pg/mL, with a sensitivity of 65.90% and a specificity of 89.80%, while the serum ALB level was 36.15 g/L, with a sensitivity of 86.40% and a specificity of 65.00%, as well as a combined sensitivity of 84.10% and a specificity of 73.90%. Serum GDF15 and serum ALB levels had a good predictive ability (AUC = 0.856, AUC = 0.887), and the ROC revealed a greater combined prediction value for the two (AUC = 0.935).

**Conclusion:**

Serum GDF15 levels could be used as a potential biomarker in the prediction of malnutrition in patients with AECOPD, offering a guidance for future clinical evaluation of malnutrition.

## Introduction

Chronic obstructive pulmonary disease (COPD) is a common respiratory disorder characterized by structural alterations in the airways and/or alveoli, caused by a prolonged exposure to harmful gases and particles, mostly from cigarette smoking, resulting in persistent respiratory symptoms and irreversible airflow limitation (1). The World Health Organization anticipates that COPD will become the third leading cause of global mortality by 2030 (2). Acute exacerbation of chronic obstructive pulmonary disease (AECOPD) usually refers to the deterioration of clinical symptoms, including increased cough and sputum production, chest tightness, and dyspnea (3). Repeated episodes of acute exacerbation cause an intensification of inflammation and a significant reduction in lung function, often resulting in rapid disease progression and increased treatment challenges. AECOPD is frequently accompanied by comorbidities such as heart failure, respiratory failure, and malnutrition (4). The prevalence of malnutrition in COPD patients has been extensively documented in numerous studies, with estimates ranging from 30 to 60% (5). The rate of hospitalization and mortality is significantly increased when coupled with malnutrition (6). The risk of death has almost tripled, particularly during acute exacerbation, profoundly impacting patient prognosis and imposing a substantial economic burden (6, 7). Therefore, early identification and prediction of malnutrition in patients with AECOPD as well as timely implementation of nutritional management strategies are crucial for optimizing clinical prognosis and reducing disease burden.

Growing evidence revealed that dietary factors, nutrients, and gut-lung axis are closely linked to the occurrence and worsening of COPD (8, 9). High intake of red or processed meat, refined grains, saturated fats and diet rich in sweets are associated with an increased risk of the occurrence of COPD (10). However, a diet rich in fruits, vegetables, whole grains, and dietary fiber is inversely associated with the decrease in lung function and risk of COPD (10), being particularly effective in smokers and ex-smokers, suggesting that healthy dietary patterns protect against the deleterious effects of smoking on lung function (11). There is an imbalance of intestinal microbiota in patients with COPD, which may be caused by the colonization of intestinal microbiota by lung microbiota or the induction of anorexia, leading to chronic inflammation and immune disorders. This phenomenon is known as “gut-lung axis” (9, 12). Gut microbiota possess anti-inflammatory effects and improve the immune defense by producing a large amount of metabolites including short-chain fatty acids through dietary fiber fermentation. Nevertheless, when the intake of healthy food is reduced in COPD patients, especially dietary fibers, the lack of fermentable dietary fiber in the gut leads to gut microbiota imbalance and affects the immune response in the lungs, promoting inflammation and malnutrition (12). Moreover, low protein intake is associated with an increased risk of mild-to-moderate COPD exacerbation and with a low body mass index (BMI) (13). However, high-protein oral nutritional supplements significantly improve the nutritional status of COPD patients and reduce the risk of death (14). This evidence shows that dietary patterns and nutritional interventions have important implications malnutrition in patients with COPD.

Malnutrition is currently recognized and mainly diagnosed according to the Global Leadership Initiative on Malnutrition (GLIM), and the European Society of Clinical Nutrition and Metabolism (ESPEN) was used as standard (15, 16). The ESPEN consensus recommends using validated screening tools to identify patients at risk for malnutrition and then diagnosing malnutrition based on a BMI of less than 18.5 kg/m^2^ or unintentional weight loss, low BMI, and low fat-free mass index (16). Similarly, the GLIM diagnostic criteria are also based on the assessment of malnutrition risk followed by a definitive diagnosis (at least one clinical presentation of unwanted weight loss, low BMI, reduced muscle mass, and one etiologic presentation of reduced food intake, inflammation, or a disease state) (15). Previous studies in patients validated the Asian BMI cutoff value of 18.5 kg/m^2^ for the diagnosis of malnutrition using the GLIM criteria (17, 18). However, BMI is often influenced by multiple factors. For example, AECOPD combined with heart failure and chronic kidney disease increases water load, resulting in body fluid overload and weight gain that does not accurately reflect nutritional status (19). The coexistence of AECOPD and heart failure activates various neurohumoral pathways in the body, including the sympathetic nervous system, renin-angiotensin-aldosterone system, and arginine vasopressor system (20). Cytokines such as arginine vasopressin, renin, and aldosterone secreted by these systems contribute to water and sodium retention in the body, leading to apparent “obesity and/or normal” presentation of patients (20). Additionally, when AECOPD is complicated by chronic kidney disease (CKD), structural or functional impairment of the kidneys results in decreased glomerular filtration rate and damage to the glomerular filtration barrier (21). This leads to a discharge of a large amount of proteins, including albumin, this phenomenon is known as the proteinuria. Since albumin maintains colloid osmotic pressure, lower serum albumin levels lead to interstitial edema (22). These conditions result in fluid overload in the body. Therefore, using BMI alone as an indicator of malnutrition for such patients may not be reliable, and additional ambulatory blood biomarkers may be beneficial. Hence, we need to identify a strategy that can be performed routinely in clinical practice, to complement the ones listed above.

Growth differentiation factor 15 (GDF15) is a member of the transforming growth factor-β superfamily with reduced expression in various human tissues, including the liver, epithelial cells, and macrophages (23). However, GDF15 expression rapidly upregulates in response to stress conditions such as inflammation or hypoxia (23). In addition to its involvement in several physiological processes including cell proliferation, differentiation, and repair, GDF15 may also play a role in various pathological processes. Elevated levels of GDF15 have been associated with cancer, cardiovascular diseases, and renal diseases, making it a potential disease biomarker (24–26). Several studies have suggested that elevated GDF15 levels in the serum are strongly associated with appetite suppression, weight loss, and muscle weakness (27, 28). Furthermore, the concentration of GDF15 in COPD patients is significantly higher than that in healthy individuals, up to 2.1 times higher (29). Moreover, GDF15 levels in AECOPD patients are higher than those in stable COPD patients and are strongly associated with reduced lung function, worsened clinical symptoms, increased frequency of acute exacerbation, and an elevated risk of mortality (29–31). Previous studies in COPD patients have associated elevated GDF15 serum levels with reduced muscle mass (32). However, it is still unknown whether serum GDF15 levels can be used to predict malnutrition in patients with AECOPD.

This study aimed to assess the predictive value of serum GDF15 for malnutrition in patients with AECOPD and to determine the optimal cut-off value for its use in clinical practice.

## Materials and methods

### Study population

A total of 142 AECOPD patients admitted to the Department of Respiratory Medicine of Anshun People’s Hospital between December 2022 and August 2023 were included in this study. Patients were divided into malnutrition (*n* = 44) and non-malnutrition (*n* = 98) group, defined as BMI < 18.5 kg/m^2^ according to the GLIM criteria. This study was approved by the Ethics Committee of the Anshun People’s Hospital (Ethics number: 2023 Specialty 1), and informed consent was obtained from all participants.

The inclusion criteria were as follows: (1) diagnosis of AECOPD according to the Global Initiative for chronic obstructive lung disease (GOLD) criteria; (2) age ≥ 40 years.

The exclusion criteria were as follows: (1) presence of bronchial asthma, pulmonary tuberculosis, bronchiectasis, or other respiratory diseases; (2) severe liver and kidney diseases, heart failure, thyroid diseases, tumors, or other conditions significantly affecting the nutritional status; (3) individuals with mental illness who could not cooperate; and (4) patients who did not complete routine blood and biochemical tests or had incomplete data after admission.

### Data collection

The following data were collected: gender, age, smoking status, BMI, mid-arm circumference (MAC), calf circumference (CC), comorbidities, nutrition risk screening 2002 (NRS2002) score, disease duration, number of acute exacerbations in the past year, COPD assessment test (CAT) score, and modified Medical Research Council (mMRC) classification. Pulmonary function tests were performed during hospitalization, including forced vital capacity (FVC), forced expiratory volume in one second (FEV_1_), and FEV_1_ as a percentage of the predicted value (FEV_1_%/pre). Patients were divided into four groups based on the global initiative for chronic obstructive pulmonary disease (GOLD) grade: grade 1 (FEV_1_%/pre ≥ 80%), GOLD grade 2 (50% ≤ FEV_1_%/pre < 80%), GOLD grade 3 (30% ≤ FEV_1_%/pre < 50%), and GOLD grade 4 (FEV_1_%/pre < 30%). BMI measurement: on the second day of admission, patients were instructed to wake up in the morning, remove any clothing or accessories that may interfere with measurements, and record their height and weight. Two measurements were taken and averaged to determine the BMI using the formula: weight (kg)/height^2^ (m^2^). MAC measurement: the circumference from the acromion on the non-dominant side to the midpoint of the olecranon line was measured twice using a tape measure with subjects’ upper limbs naturally hanging down. The average values were calculated. CC measurement: two measurements were taken using a tape measure around the broadest part of their non-dominant lower leg after allowing the subjects to stand naturally with their legs shoulder-width apart. The average values were calculated.

Fasting peripheral venous blood was collected from all subjects to detect serum GDF15 levels. The supernatant was separated by centrifugation at 2,000 RPM for 20 min after natural coagulation at room temperature and stored in an ultra-low temperature refrigerator at −80 °C until further use. Serum GDF15 levels were measured using a GDF-15 Kit (Jiangsu Jingmei Biotechnology Co., Ltd., Jiangsu, China) following the manufacturer’s instructions.

### Statistical analysis

Statistical analysis was performed using SPSS 26.0 for Windows (IBM Corporation, Armonk, NY, United States). Normally distributed data were presented as mean ± SD, and the *t*-test was used for group comparisons. Non-normally distributed data, median, and interquartile range were used, and the Mann-Whitney U test was used for group comparisons. Count data are expressed as percentages (%), and the chi-square test was used for group comparisons.

A Spearman correlation analysis was performed to investigate the relationship between serum GDF15 levels, baseline data, and clinical indicators. The binary logistic regression analysis was used to identify independent risk factors for malnutrition in patients with AECOPD. A receiver operating characteristic (ROC) curve analysis evaluated the predictive value of serum GDF15, serum ALB, and their combination in identifying malnutrition among AECOPD patients by calculating the area under the curve (AUC). The significance level was set at *p* < 0.05.

## Results

### Baseline characteristics of subjects

This study included 142 patients and their baseline characteristics are shown in Table 1. Overall, the mean age of all patients with AECOPD was 67.67 ± 8.89 years and included 120 males and 22 females, with a malnutrition prevalence rate of approximately 30.99%. Next, we analyzed the differences between patients with and without malnutrition. MAC and CC were significantly lower in patients with malnutrition and AECOPD, as shown in Table 1. In terms of clinical symptoms, the CAT score and mMRC classification were higher in patients with malnutrition and AECOPD. In addition, the GOLD grade was significantly increased in patients with malnutrition and AECOPD.

**TABLE 1:** Baseline characteristics of the included subjects.

Variables	Malnutrition (*n* = 44)	Non-malnutrition (*n* = 98)	*p*-value
Gender			0.191
Male	39 (88.6)	78 (79.6)	
Female	5 (11.4)	20 (20.4)	
Mean age, years	68.27 ± 7.43	68.19 ± 8.37	0.957
BMI, kg/m^2^	18.269 (17.56, 18.43)	22.63 (20.25, 25.80)	< 0.001
MAC, cm	24.80 (23.85, 27.58)	29.50 (27.25, 32.03)	< 0.001
CC, cm	30.31 ± 2.93	33.52 ± 3.18	< 0.001
Index of smoking, pack-years	49.00 (39.00, 60.00)	45.50 (0.00, 62.50)	0.240
**Comorbidity**			
Hypertension	12 (27.3)	25 (25.5)	0.825
Coronary heart disease	2 (4.5)	5 (5.1)	0.887
Diabetes mellitus	3 (6.8)	9 (9.2)	0.639
Respiratory failure	26 (59.1)	42 (42.9)	0.073
Number of acute exacerbations in the past year, times	1.5 (1.00, 2.00)	1.00 (0.00, 2.00)	0.082
Course of disease, years	8.00 (3.13, 14.50)	5.75 (3.00, 11.00)	0.212
NRS2002 score	6.00 (5.00, 7.00)	3.00 (2.00, 3.25)	< 0.001
CAT score	22.93 ± 4.73	16.76 ± 5.78	< 0.001
mMRC classification	4 (3, 4)	3 (2, 3)	< 0.001
GOLD grade	4 (3, 4)	3 (2, 4)	0.001

Data are expressed as mean ± SD, median (interquartile range), or frequency (%). BMI, body mass index; MAC, mid-arm circumference; CC, calf circumference; NRS2002 score, nutritional risk screening 2002; CAT score, COPD assessment test; mMRC classification, modified medical research council; GOLD grade, global initiative for chronic obstructive pulmonary disease.

### Serum GDF15 levels and clinical indicators of subjects

Table 2 shows that patients with malnutrition had higher GDF15 levels in the serum and C-reactive protein (CRP) than patients without malnutrition, while total protein (TP) and ALB levels, as well as the prognostic nutritional index (PNI) were lower in patients with malnutrition. The differences were statistically significant. Patients with and without malnutrition showed no significant differences in hemoglobin, neutrophil/lymphocyte ratio (NLR), lymphocyte/monocyte ratio (LMR), and platelet/lymphocyte ratio (PLR).

**TABLE 2:** Serum GDF15 and clinical indicators of subjects.

Clinical indicators	Malnutrition (*n* = 44)	Non-malnutrition (*n* = 98)	*p*-value
GDF15, pg/mL	1,117.47 ± 93.00	951.61 ± 128.15	< 0.001
CRP, mg/L	22.13 (11.24, 55.89)	12.05 (3.52, 38.86)	0.002
TP, g/L	65.70 (64.50, 66.80)	68.80 (67.40, 69.83)	< 0.001
ALB, g/L	35.10 (34.25, 35.90)	37.00 (36.20, 38.60)	< 0.001
PNI	39.80 (38.14, 42.01)	42.65 (40.76, 45.99)	< 0.001
Hemoglobin, g/L	134.05 ± 22.09	141.28 ± 25.32	0.104
NLR, %	5.54 (3.43, 8.56)	4.69 (3.00, 7.36)	0.320
LMR, %	2.07 (1.48, 3.26)	2.39 (1.59, 3.26)	0.059
PLR, %	175.74 (112.9, 227.28)	142.90 (104.38, 216.48)	0.180

Data are expressed as mean ± SD, median (interquartile range). GDF15, growth differentiation factor 15; CRP, C-reactive protein; TP, total protein; ALB, albumin; PNI, prognostic nutritional index, albumin+5*lymphocyte count; NLR, neutrophil/lymphocyte ratio; LMR, lymphocyte t/monocyte ratio; PLR, platelet/lymphocyte ratio.

### Serum GDF15 levels and baseline characteristics and clinical indicators

Serum GDF15 levels were significantly negatively correlated with BMI (*r* = −0.562, *p* < 0.001), MAC (*r* = −0.505, *p* < 0.001), CC (*r* = −0.490, *p* < 0.001), TP (*r* = −0.486, *p* < 0.001), ALB (*r* = −0.445, *p* < 0.001), and PNI (*r* = −0.276, *p* = 0.001). This was confirmed by the positive correlation between serum GDF15 levels, CAT score (*r* = 0.286, *p* = 0.001), mMRC classification (*r* = 0.310, *p* < 0.001), and GOLD grade (*r* = 0.177, *p* = 0.035). The results are presented in Table 3.

**TABLE 3:** Spearman correlation analysis between serum GDF15 levels and baseline data and clinical indicators.

Variables	GDF15, pg/mL	
	*r*	*p*-value
BMI, kg/m^2^	−0.562	< 0.001
MAC, cm	−0.505	< 0.001
CC, cm	−0.490	< 0.001
TP, g/L	−0.486	< 0.001
ALB, g/L	−0.445	< 0.001
CRP, mg/L	0.318	< 0.001
PNI	−0.276	0.001
CAT score	0.286	0.001
mMRC classification	0.310	< 0.001
GOLD grade	0.177	0.035

BMI, body mass index; MAC, mid-arm circumference; CC, calf circumference; TP, total protein; ALB, albumin; CRP, C-reactive protein; PNI, prognostic nutritional index, albumin+5*lymphocyte count; CAT score, COPD assessment test; mMRC classification, modified medical research council; GOLD grade, global initiative for chronic obstructive pulmonary disease.

### Risk factors for malnutrition in AECOPD patients

After performing a binary logistic regression analysis, we used non-malnutrition and malnutrition as dependent variables (non-malnutrition = 0, malnutrition = 1), and the indicators with statistically significant differences in the text as independent variables. Table 4 illustrates that serum GDF15 levels (OR = 1.010, 95% CI, 1.003∼1.016) independently contributed to the risk of malnutrition in patients with AECOPD.

**TABLE 4:** Binary logistic regression analysis of risk factors in patients with malnutrition and AECOPD.

Variables	β	SE value	Wald value	*p*-value	OR	95% CI
MAC, cm	−0.573	0.298	3.683	0.055	0.564	(0.314∼1.012)
CC, cm	0.464	0.276	2.817	0.093	1.590	(0.925∼2.733)
TP, g/L	−0.189	0.317	0.357	0.550	0.827	(0.445∼1.540)
ALB, g/L	−0.901	0.543	2.751	0.097	0.406	(0.140∼1.178)
CRP, mg/L	−0.002	0.006	0.084	0.772	0.998	(0.986∼ 1.011)
PNI	0.105	0.132	0.631	0.427	1.111	(0.857∼1.440)
GDF15, pg/mL	0.010	0.003	9.174	0.002	1.010	(1.003∼1.016)
CAT score	0.111	0.094	1.404	0.236	1.118	(0.930, 1.343)
mMRC classification	0.198	0.842	0.055	0.814	1.219	(0.234, 6.346)
GOLD grade	0.083	0.551	0.022	0.881	1.086	(0.369, 3.199)

MAC, mid-arm circumference; CC, calf circumference; TP, total protein; ALB, albumin; CRP, C-reactive protein; PNI, prognostic nutritional index, albumin+5*lymphocyte count; GDF15, growth differentiation factor 15; CAT score, COPD assessment test; mMRC classification, modified medical research council; GOLD grade, global initiative for chronic obstructive pulmonary disease.

### Predictive value of serum GDF15 levels, serum ALB levels and their combination in malnutrition patients with AECOPD

**FIGURE 1:**
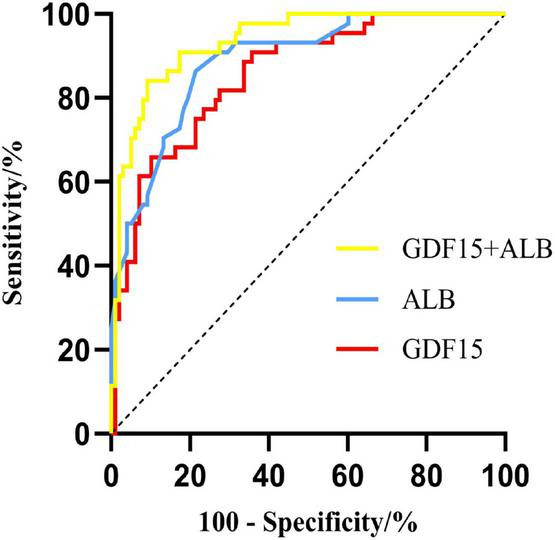
ROC curves of serum GDF15 levels and albumin levels, and their combination for predicting malnutrition in AECOPD patients.

The optimal cut-off value of serum GDF15 levels and serum ALB levels for predictive malnutrition in AECOOPD patients was 1,092.885 pg/mL and 36.15 g/L, respectively, with a sensitivity of 65.90 and 86.40%, respectively, as well as specificity of 89.80 and 65.00%, respectively. The combination had a sensitivity of 84.10% and specificity of 73.90%. The AUC of serum GDF15 levels was 0.856 (95% CI, 0.791∼0.920) and the AUC of serum ALB levels was 0.887 (95% CI, 0.831∼0.943), and the combination AUC was 0.935 (95% CI, 0.895∼0.975). These results demonstrated that the combined detection had a stronger predictive ability for malnutrition in AECOPD patients. The results are presented in Table 5 and Figure 1.

**TABLE 5:** Predictive value of serum GDF15 levels, serum ALB levels and their combination in malnutrition patients with AECOPD.

Indicator	AUC	95% CI	Cut-off value	Sensitivity/%	Specificity/%	Youden index	*p*-value
GDF15 (pg/mL)	0.856	(0.791∼0.920)	1,092.885	65.90	89.80	0.557	< 0.001
ALB (g/L)	0.887	(0.831∼0.943)	36.15	86.40	65.00	0.514	< 0.001
GDF15+ALB	0.935	(0.895∼0.975)	–	84.10	73.90	0.58	< 0.001

GDF15, growth differentiation factor 15; ALB, albumin; GDF15+ALB, growth differentiation factor 15+albumin.

## Discussion

In this study, we examined the levels of serum GDF15 in AECOPD patients with and without malnutrition and the differences between the two groups were compared. We found a significant increase in serum GDF15 levels in AECOPD patients with malnutrition, which was negatively correlated with nutritional indicators such as BMI, MAC, CC, TP, ALB, and PNI, while demonstrating a positive correlation with the inflammatory marker CRP. Furthermore, serum GDF15 levels were positively correlated with CAT score, mMRC classification, and GOLD grade. Although our findings are mainly consistent with previous research reports, several new discoveries have emerged: (1) serum GDF15 is an independent risk factor for AECOPD patients with comorbid malnutrition; (2) serum GDF15 levels hold promise as a predictive biomarker for identifying malnutrition during AECOPD episodes, with the combination of serum ALB levels having a higher predictive ability for malnutrition in patients with AECOPD.

In clinical practice, BMI is commonly used as an initial assessment tool to evaluate the nutritional status of patients. However, it should be emphasized that BMI is influenced by various factors and may not accurately reflect an individual’s actual nutritional status. Studies have demonstrated that even when COPD patients are within the normal BMI range, they often exhibit significant muscle quality decline and dysfunction, both being signs of malnutrition (32). These conditions can cause respiratory muscle weakness and exacerbate the progression of COPD. Furthermore, in patients with AECOPD who have comorbidities such as heart failure or chronic kidney disease, fluid overload can result in weight gain and falsely increased BMI levels, overestimating their actual nutritional status (19). Therefore, relying solely on BMI to diagnose malnutrition may result in an inaccurate assessment due to its susceptibility to diseases. To evaluate the nutritional status, especially among AECOPD patients, patients with severe liver, and kidney diseases as well as heart failure were excluded in our study, even though a BMI < 18.5 kg/m^2^ was the criteria for a diagnosis of malnutrition according to GLIM standards. The findings revealed a malnutrition prevalence rate of approximately 30.99% in our patient population, which was consistent with previous research results. Therefore, it is imperative to conduct comprehensive evaluations considering the potential coexisting medical conditions when using BMI alone for the clinical diagnosis of malnutrition.

ALB is a widely used clinical indicator for malnutrition assessment. This study revealed that AECOPD patients with malnutrition had lower serum ALB levels than those without malnutrition. Moreover, serum ALB levels show high predictive efficiency for malnutrition in AECOPD patients. Nevertheless, a binary logistic regression analysis showed that the serum albumin levels were not an independent risk factor for malnutrition in AECOPD patients. This finding might be related to the small sample size included in this work. Therefore, in the future, we will perform a large sample, multicenter prospective study to further validate the results. Moreover, the average half-life of albumin is approximately 20 days (33). Patients with AECOPD often experience reduced substance intake and increased catabolism, leading to potential lagging phenomena that reflect rapid changes in nutritional status. Serum GDF15 levels can rapidly increase under inflammatory conditions, with a half-life of approximately 3 h (34), and they are also linked to weight loss and weakness. These findings suggested that serum GDF15 levels might serve as a more effective indicator of nutritional status than serum ALB levels in critically ill patients in a short time. Furthermore, in AECOPD patients with comorbidities, such as heart failure or chronic renal failure, serum albumin might be lost through urinary excretion, resulting in hypoalbuminemia (33). One of the roles of serum albumin is to maintain plasma colloid osmotic pressure. Hypoalbuminemia may lead to fluid overload and promote weight gain, which is detrimental to the early detection of malnutrition (22, 33). Therefore, a comprehensive evaluation incorporating multiple factors is necessary to accurately assess the nutritional condition.

GDF15 belongs to the transforming growth factor-β superfamily and its expression is typically low in the human body under physiological conditions (23). However, its expression rapidly increases during inflammation and hypoxia and often serves as an inflammatory marker during the acute phase. Studies have revealed a significant increase of GDF15 levels among patients with COPD and AECOPD than those in good health, which is strongly associated with disease prognosis. Furthermore, high GDF15 levels have been observed in patients with old age and chronic kidney disease, and are closely linked to nutritional status (35, 36). Previous research has also demonstrated a strong correlation between GDF15 levels and muscle quality in patients with COPD. When muscle quality declines and dysfunction occurs in COPD patients, the respiratory muscles weaken, resulting in increased airway obstruction and more respiratory effort during static states. This results in significant energy expenditure and malnutrition in some individuals. Currently available methods for assessing muscle quality and function include magnetic resonance imaging (MRI), dual-energy X-ray absorptiometry (DEXA), and bioelectrical impedance analysis (BIA). However, it is difficult to perform procedures due to high costs, specific equipment, and specialized training. Clinicians commonly employ MAC and CC measurements as preliminary indicators of muscle quality evaluation (37). The results of this study also confirmed a significant negative correlation between serum GDF15 levels and TP, ALB, and PNI, particularly with BMI (*r* = −0.562, *p* < 0.001), MAC (*r* = −0.505, *p* < 0.001), and CC (*r* = −0.490, *p* < 0.001). This indicated that GDF-15 was involved in malnutrition in patients with AECOPD. Furthermore, serum GDF15 levels were significantly increased in AECOPD patients with malnutrition and were identified as an independent risk factor for AECOPD in patients with malnutrition. In this study, we separately analyzed the predictive efficacy of serum GDF-15 and albumin levels in patients with malnutrition and AECOPD. ROC analysis showed that serum GDF-15 levels had a lower sensitivity than serum albumin, but a higher specificity in predicting malnutrition in patients with AECOPD. Using this indicator alone with serum GDF15 levels to assess malnutrition might result in false negative results because serum GDF-15 levels might increase with age (38). Therefore, serum GDF15 levels combined with serum albumin levels were used to predict AECOPD malnutrition in patients, and the findings revealed a high sensitivity and specificity, with an AUC of 0.935. This indicated that the combined detection had a greater predictive ability for malnutrition in AECOPD patients.

The underlying mechanism by which GDF15 contributes to malnutrition in AECOPD remains unclear. It is possible that GDF15 may contribute to the development of malnutrition by activating signal transduction pathways and interacting with neurotrophic factors (39–41). GDF15 reduces body weight by inhibiting food intake, and its effect is mediated by its binding with its receptor glial cell derived neurotrophic factor receptor alpha like (GFRAL). GFRAL belongs to the family of glial cell-derived neurotrophic factor receptors, which are mainly present in neurons located in the nucleus tractus solitaris region of the posterior brain and the posterior region of the brainstem, which are the centers for the induction of vomiting and/or anorexia. About the main mechanism for anorexia response may be GDF15 combined with GFRAL specificity, leads to the activation and phosphorylation of the Ret signal transduction pathway, and activation of signal transduction molecules such as Akt, Erk and PLC (42). It has also been found that GFRAL is localized on cholecystokinin (CCK) positive neurons; GDF15 activates CCK neurons, and GDF15-induced anorexia is attenuated by CCK signaling blockade, suggesting that GDF15 is mediated by anorexigenic signaling in CCK neurons in the brainstem (43). GDF15 also reduces food intake by delaying gastric emptying through the vagus nerve, and this change is abolished after bilateral vagotomy (34). Borner (44) found a significant difference in the effect of delayed gastric emptying only after the treatment with high concentrations of GDF15, suggesting that this effect requires high levels of GDF15 to be effective. It may also be associated with the inflammatory response observed in AECOPD patients, as GDF15 levels are significantly elevated in these conditions. Furthermore, this study discovered a correlation between GDF15 and CRP levels. CRP is an acute-phase protein synthesized by the liver and is widely used as an early, sensitive indicator of inflammation (45). Elevated CRP levels hinder nutritional support efficacy also contributing to the early detection of malnutrition (45). Hypoxia exacerbates gastrointestinal congestion, resulting in reduced intake among patients, increased metabolism, and a negative nitrogen balance, all contributing to malnutrition (46). Therefore, when GDF15 levels are elevated in AECOPD patients with concurrent malnutrition, potential targeted anti-inflammatory interventions may improve malnutrition.

This study has certain limitations. Firstly, it is important to acknowledge that this study lacked a control group of individuals who were in good health and had stable COPD conditions. It should be also noted that this was an observational study with a relatively small sample size, and no additional research was performed on serum GDF15 levels and the prognosis of patients with AECOPD malnutrition, for example, hospitalization duration, hospitalization expenses, mortality, and acute exacerbation time should be considered the next time. To validate the reliability of serum GDF15, we need to conduct more prospective, multi-center investigations in the future. Furthermore, a recent study has shown that constructing the mesoporous PdPt-assisted LDI MS for biomarker analysis results in a more accurate early diagnosis of COPD and AECOPD (47). In the future, we may be able to use this platform to detect serum biomarkers, such as GDF15 and ALB to identify malnutrition in patients with AECOPD to provide early nutritional therapy.

Accordingly, we hypothesized that serum GDF15 might be used as a potential biomarker to detect malnutrition in patients with AECOPD.

## Conclusion

Serum GDF15 levels could be used as a potential diagnostic biomarker for predicting malnutrition in patients with AECOPD, providing direction for future clinical evaluations of malnutrition.

## Data availability statement

The original contributions presented in this study are included in this article/supplementary material, further inquiries can be directed to the corresponding authors.

## Ethics statement

The studies involving humans were approved by the Medical Ethics Committee of Anshun People’s Hospital. The studies were conducted in accordance with the local legislation and institutional requirements. The participants provided their written informed consent to participate in this study.

## Author contributions

GS: Data curation, Formal analysis, Investigation, Writing – original draft. LY: Data curation, Investigation, Writing – review & editing. ZT: Data curation, Writing – review & editing. YW: Data curation, Writing – review & editing. XH: Data curation, Formal analysis, Writing – review & editing. YT: Data curation, Formal analysis, Funding acquisition, Investigation, Project administration, Supervision, Writing – review & editing.
